# SG-APSIC1084: Reduce cost and resterilization rate of reusable medical device sets by reorganizing and rearranging packaging and process

**DOI:** 10.1017/ash.2023.95

**Published:** 2023-03-16

**Authors:** Rungtawan Sutthiwichienchot

**Affiliations:** Central Sterilizing Services Association, Bankok, Thailand

## Abstract

**Objectives:** We evaluated the resterilization rate, user satisfaction, and cost of resterilization after rearranging and packing of reusable instrument sets. **Methods:** For 1 month in July 2018, we conducted an observational prospective study in 39 service departments for which sterilization and instrument packing was done by the central sterile supply department (CSSD). Common sterile instrument sets (eg, intercostal drainage (ICD) sets, bone-marrow aspiration sets, or suture sets) were analyzed to set up basic surgical instruments for common procedures and specific instruments for each procedure. Sets for common procedures were then packed and rearranged for use universally in various procedures separately from specific instruments. A questionnaire survey was delivered to all 39 service departments to evaluate user satisfaction. The resterilization rates and cost analyses before and after the rearranging and packing were compared for their effectiveness. The data were analyzed using descriptive statistics for percentage, mean, standard deviation, and inferential statistics. Categorical data were analyzed using the χ^2^ test and continuous data were analyzed using a *t* test with significance level of 0.05. **Results:** The resterilization rate decreased significantly from 7.1% to 0.1%. The cost of resterilization decreased from 76,500 Thai baht (US $2,287) to 4,800 Thai baht (US $143) within 1 month. Overall, user satisfaction regarding this intervention was 85.2%. **Conclusions:** This study highlights the need for the evaluation of process and customer demand to improve user satisfaction and reduce hospital cost by customizing the sterilization packaging and rearranging process.

